# Der p 23, A Frequent IgE Sensitizer in Humans, Induces Airway Inflammation in Mice

**DOI:** 10.3390/ijms262110765

**Published:** 2025-11-05

**Authors:** Karen Donado, Luis Durango, Inés Benedetti, Nikolina Todorovic, Ronald Regino, Ana Lozano, Victoria Marrugo, Randy Reina, Dilia Mercado, Nathalie Acevedo, Josefina Zakzuk, Enrique Fernández-Caldas, Walter Keller, Leonardo Puerta, Luis Caraballo

**Affiliations:** 1 Institute for Immunological Research, University of Cartagena, Cartagena 130014, Colombia; kdonador@unicartagena.edu.co (K.D.); ldurangon@unicartagena.edu.co (L.D.); alozanom@unicartagena.edu.co (A.L.); vmarrugod@unicartagena.edu.co (V.M.); rreinar@unicartagena.edu.co (R.R.); dmercadob@unicartagena.edu.co (D.M.); nacevedoc@unicartagena.edu.co (N.A.); jzakzuks@unicartagena.edu.co (J.Z.); lcaraballog@unicartagena.edu.co (L.C.); 2 Department of Basic Sciences, School of Medicine, University of Cartagena, Cartagena 130014, Colombia; ebenedettip1@unicartagena.edu.co; 3 Institute of Molecular Biosciences, Structural Biology and Biophysics, University of Graz, 8010 Graz, Austriawalter.keller@uni-graz.at (W.K.); 4 Division of Allergy and Immunology, Department of Internal Medicine, University of South Florida Morsani College of Medicine, Tampa, FL 33613, USA; enricaldas@telefonica.net

**Keywords:** house dust mite, Der p 23, animal model, IgE-sensitization, asthma, airway inflammation, allergy

## Abstract

Der p 23 induces a high-frequency sensitization in allergic individuals. However, its allergenic activity and clinical impact are scarce. We aimed to evaluate the ability of rDer p 23 to induce allergic inflammation in a mouse model and to test IgE reactivity in humans. Female Balb/c mice were sensitized and challenged with rDer p 23 and *Dermatophagoides pteronyssinus* extract. Specific antibodies were determined by ELISA, inflammatory cell infiltration and goblet cells hyperplasia were evaluated by lung histology, and bronchial hyperreactivity (BHR) was assessed by the FinePoint RC System^TM^ and whole-body plethysmography (WBP). IgE reactivity was evaluated by ELISA, the basophils activation test (BAT) and the skin pick test (SPT) in humans. rDer p 23, produced in *Escherichia coli*, adopts a random coil structure, predominantly exists in a monomeric state, and exhibits high stability. rDer p 23-treated mice showed a significant increase in lung resistance and bronchial hyperreactivity, as well as in eosinophils, neutrophils, and T cell count in bronchoalveolar lavage fluid (BALF). Cytokine and antibodies profiles were biased to a Type-2 response. No significant difference was observed in group 2 Innate Lymphoid Cells (ILC-2s) in lung and regulatory T cells (Treg) in the spleen. In asthmatic individuals sensitized to *D. pteronyssinus*, serum IgE reactivity to rDer p 23 was 67.5%. BAT and SPT results were significantly higher in allergic patients. Our findings support the pro-allergenic role of rDer p 23 in the development of the pathological features of asthma.

## 1. Introduction

Allergic diseases are a major health problem with prevalence reaching up to 30% in some countries around the world [1,2]. The prevalence of asthma in tropical regions is as high as that observed in temperate countries, but with more notable epidemiological differences, such as perennial exposure to house dust mite (HDM) aeroallergens, the high frequency of helminth infections in the population, socio-economic conditions, and genetic factors [3,4,5]. Asthma develops due to repeated exposure to HDM allergens. *D. pteronyssinus* and *Blomia tropicalis* proliferate more rapidly in warm and humid climates, which explains their almost exclusive distribution, and are the main sensitizers in tropical environments [6,7,8]. Sensitization to these two mite species is highly prevalent among patients with asthma in tropical regions, with rates exceeding 90% in some studies [7].

Molecular allergology research has provided valuable insights into the structure and function of individual allergenic molecules, leading to the development of methodologies that incorporate allergens relevant to specific populations. These approaches promise more reliable and precise diagnosis and treatment in affected individuals. Tools such as Component-Resolved Diagnosis (CRD) and Allergen-Specific Immunotherapy (AIT) based on purified allergens allow the identification of specific proteins responsible for the disease and, consequently, improve asthma management [9,10].

Currently, it is well accepted that, for an appropriate characterization, both the frequency of binding to specific IgE antibodies and the ability to induce an inflammatory process are needed [11,12]. Knowledge of the allergenic activity of HDM allergens provides valuable insights into the pathogenesis of asthma [13]. However, this information remains limited in tropical regions and other parts of the world, where the absence of a comprehensive catalog of clinically relevant allergens hampers the management of affected individuals.

Among *D. pteronyssinus* allergens, Der p 1, Der p 2, and Der p 23 are the most prevalent. They have been strongly associated with both childhood and adult asthma [14,15,16], and are considered clinically relevant. However, unlike Der p 1 and Der p 2, whose mechanisms of action in allergic inflammation are well studied [17,18,19,20,21,22], little is known about the inflammatory properties of Der p 23 and its capacity to stimulate the innate and adaptive immune response [12,23]. In fact, the role of innate cells and their mediators as key mechanisms for activating pathways associated with allergic inflammation is under study [24]. ILC-2s represent a subset of cells that can induce a proinflammatory process when stimulated by alarmins and other molecules produced by the epithelium [25,26].

Structural and sequence analysis of Der p 23 shows homology with peritrophins, proteins that contain chitin-binding domains and are part of the peritrophic matrix that lines the arthropod intestine and fecal pellets [27,28]. In addition to its high frequency of sensitization, Der p 23 is clinically associated with the presentation of asthma [15,29,30] and activates basophils in vitro through IgE-dependent mechanisms [28]. Therefore, defining the role of this allergen in the inflammatory allergic response will contribute to a better understanding of its basic and clinical impacts.

## 2. Results

### 2.1. rDer p 23 Was Produced with a High Degree of Stability and Purity

**Figure 1:**
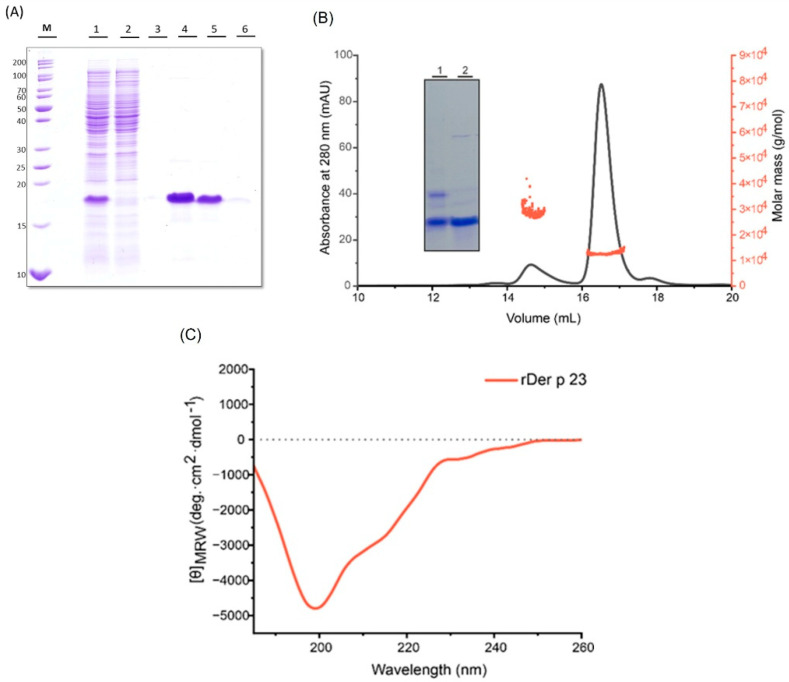
Purification and biophysical characterization of rDer p 23. (**A**) Commassie blue stained 15% (*w/v*) SDS-PAGE shows purification using NiNTA affinity column. The protein in the lysate (lane 1) was captured by the resin (flow through, lane 2) and eluted with Native Elution Buffer (NEB) (lanes 3–5). (**B**) SEC-MALS of rDer p 23. Absorbance (mAU) recorded at 280 nm (*y*-axis; black) shows the elution peaks, whereas molar mass (*y*-axis, orange) depicts the molecular weight distribution across the peak. Inset with a Commassie blue stained 12% (*w*/*v*) SDS polyacrylamide gel of purified rDer p 23. Lane 1-rDer p 23 under non-reducing conditions, lane 2-rDer p 23 under reducing conditions. (**C**) CD analysis of rDer p 23 (orange). The mean residue molar ellipticities (*y*-axis: degree cm^2^ dmol^−1^) were recorded in the wavelength range from 185 to 260 nm (*x*-axis).

rDer p 23 was expressed using Isopropyl-1-Beta-thiogalactopyranoside (IPTG) in *E. coli* as a His-tagged protein purified by affinity chromatography with Ni-NTA, using an NEB (Figure 1A). Size exclusion chromatography coupled to multi-angle light scattering (SEC-MALS) was used to evaluate the oligomeric state and polydispersity. At a concentration of 2 mg/mL, rDer p 23 predominantly exists as a monomer in solution, with an estimated molecular weight of 12 kDa (± 12.7%), as determined by the MALS detector, which is in large agreement with the theoretically calculated molecular weight (10.5 kDa). Additionally, a minor fraction was detected that might correspond to an oligomeric form (~28 kDa) (Figure 1B). SDS-PAGE analysis under reducing conditions indicates that the protein exists as a monomer, while its migration pattern under non-reducing conditions confirms the presence of the minor oligomeric fraction. When the sample was treated with the reducing agent TCEP∙HCl (Tris (2-carboxyethyl) phosphine hydrochloride), the oligomeric form was no longer detectable, suggesting that its formation is likely mediated by intermolecular disulfide bond formation (Figure 1B). Upon storage for four weeks at 4 °C, the protein showed high stability, as no significant change was observed in the gel under reducing conditions (Figure S1). Circular dichroism (CD) spectroscopy of rDer p 23 revealed a minimum at 200 nm, suggesting that the protein adopts a random coil secondary structure formation (Figure 1C, Table S1).

### 2.2. rDer p 23 Induced High Bronchial Hyperresponsiveness and Airway Resistance in the Mouse Model

**Figure 2:**
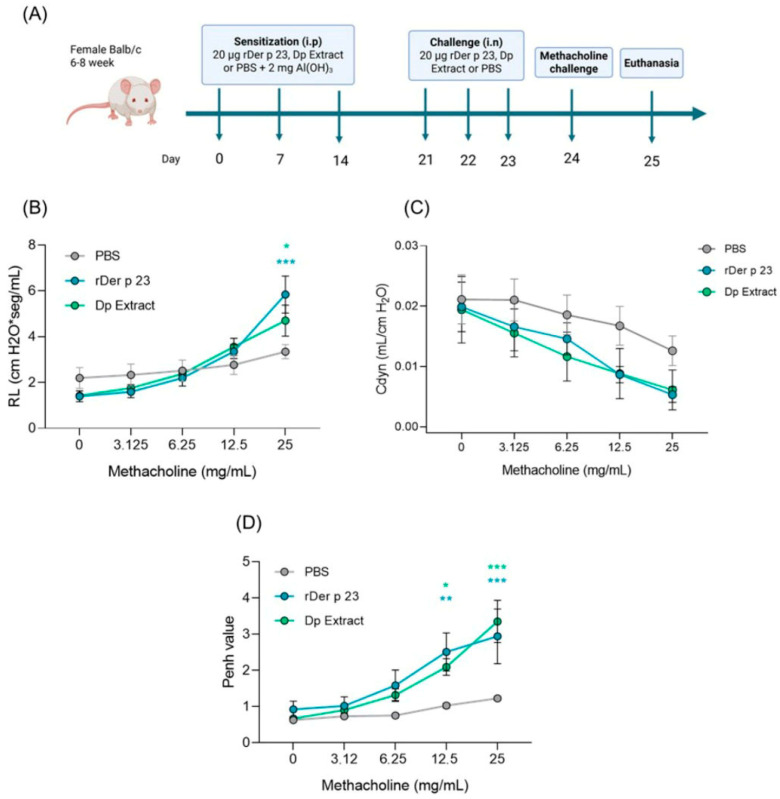
Allergic airway inflammation model and response to methacholine challenge in Balb/c mice. (**A**) Six- to eight-week-old female Balb/c mice were sensitized by intraperitoneal (i.p.) injections on days 0, 7, and 14, followed by daily intranasal (i.n.) challenges on days 21, 22, and 23 with rDer p 23. On day 24, the reactivity to methacholine challenge was performed followed by euthanasia on day 25. The same model was carried out in PBS- and *D. pteronyssinus* extract-treated mice as negative and positive controls, respectively. (**B**,**C**) Airway hyperreactivity to methacholine challenge was evaluated in tracheotomized mice by FinePoint RC System^TM^ RL (**B**) and Cdyn (**C**). (**D**) Methacholine challenge by WPB. Data are shown as mean (*n* = 4–6 mice) and the bars representing the standard error of the mean (SEM). These results were from two independently replicated experiments. Two-way ANOVA with Tukey’s post hoc analysis was applied to compare data between groups. * *p* < 0.05, ** *p* < 0.01, *** *p* < 0.001.

Administration of Der p 23 in the mouse model (Figure 2A) had a significantly greater effect on methacholine-induced bronchial hyperactivity. Lung resistance (RL) in tracheotomized mice treated with rDer p 23 and *D. pteronyssinus* extract was significantly higher at the 25 mg/mL dose of metacholine compared to mice treated with PBS (Figure 2B). In addition, dynamic compliance (Cdyn) decreased when the methacholine dose increased, indicating lower airway distensibility; however, no statistically significant changes were found between the groups evaluated (Figure 2C). WBP showed that Penh values in mice treated with rDer p 23 and *D. pteronyssinus* extract at concentrations of 12.5 and 25 mg/mL were significantly higher compared to those in mice treated with PBS (Figure 2D). These data indicate increased bronchoconstriction induced by exposure to the purified allergen or the allergenic extract.

### 2.3. rDer p 23 Induced Tissue Inflammation and Hypersecretion of Mucus in the Airways

**Figure 3:**
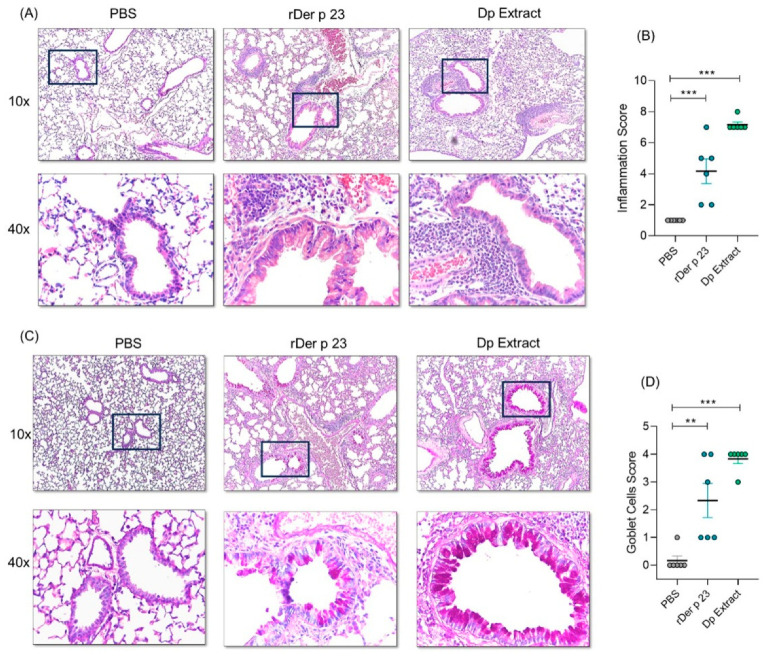
Lung histology analysis. (**A**) Representative images in 10X (top) and 40X (below) magnification of hematoxylin-eosin (H/E)-stained lung sections from PBS-, rDer p 23-, and *D. pteronyssinus* extract-treated mice. (**B**) Statistical analysis of inflammation score of H/E-stained lung histological sections. (**C**) Representative images in 10X (top) and 40X (below) a blank square magnification for periodic acid–Schiff (PAS)-stained lung sections from PBS-, rDer p 23-, and *D. pteronyssinus* extract-treated mice. PAS-positive goblets cells are identified by magenta staining. (**D**) Statistical analysis of goblet cell score of PAS-stained lung sections. Data are shown as mean (*n* = 6 mice) and the bars represent the SEM. These results were from two independently replicated experiments. One-way ANOVA with Dunnett’s post hoc analysis was applied to compare data between groups. ** *p* < 0.01, *** *p* < 0.001.

Lung histological analysis of mice treated with rDer p 23 and *D. pteronyssinus* extract showed a tissue inflammatory response with a higher content of peribronchial and perivascular infiltrates compared to mice treated with PBS (Figure 3A). Lung inflammation induced by *D. pteronyssinus* extract was also significantly higher than that which was induced by PBS treatment. The inflammation score with rDer p 23 was lower than with the *D. pteronyssinus* extract (Figure 3B). In addition, a significant hyperplasia of goblet cells was observed in mice treated with rDer p 23 and *D. pteronyssinus* extract compared to that which was in mice treated with PBS (Figure 3C). The goblet cells score was significantly higher in mice treated with rDer p 23 and *D. pteronyssinus* extract compared to that which was in mice treated with PBS (Figure 3D). These results show that mucus production and the inflammatory response to the individual allergen are similar to those which are induced by the complete extract.

### 2.4. rDer p 23 Recruits Proinflammatory Cells into Mouse Airway

**Figure 4:**
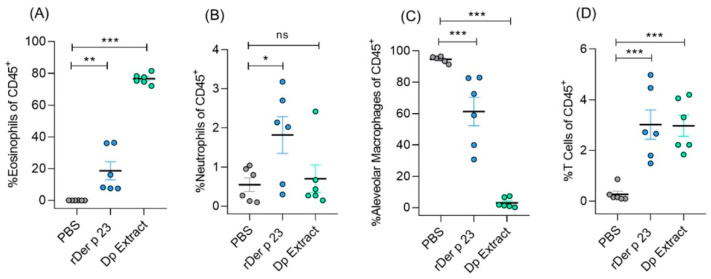
Analysis of immune cell population in bronchoalveolar lavage fluid. Statistical analysis of percentage of eosinophils (**A**), neutrophils (**B**), alveolar macrophages (**C**), and T cells of total CD45^+^ cells (**D**) in BALF of PBS-, rDer p 23-, and *D. pteronyssinus* extract-treated mice. The mean value of each group (*n* = 6 mice) and the bars representing the SEM of the mean are shown. These results were from two independently replicated experiments. The comparison was performed using the one-way ANOVA test and Dennett’s post hoc analysis. * *p* < 0.05, ** *p* < 0.01, *** *p* < 0.001, ns: not significant.

Flow cytometry analysis of BALF cellular content showed that rDer p 23-treated mice had an increased recruitment of immune cells, mainly eosinophils (~20%) (Figure 4A), and to a lesser extent neutrophil (~2%) (Figure 4B). These percentages were significantly higher in rDer p 23-treated mice compared to PBS-treated mice. Furthermore, a significant decrease in the percentage of alveolar macrophages was found in mice treated with rDer p 23 and *D. pteronyssinus* extract (Figure 4C). The percentage of T cells in BALF was significantly higher in mice treated with rDer p 23 and *D. pteronyssinus* extract (Figure 4D).

### 2.5. rDer p 23 Did Not Induce Significant Changes in ILC-2 Frequency in the Lung

We identified ILC-2s (Lin^-^CD90.2^+^CD127^+^ST2^+^) in mouse lung samples based on the gating strategy shown in Figure S2. Although no statistically significant differences were found in the percentage of ILC-2s between the groups tested, it was observed that immunized mice with rDer p 23 showed a higher, but non-significant, tendency toward a population of total ILCs (Lin^-^CD90.2^+^).

### 2.6. rDer p 23 Induced Humoral Response of Type-2 Profile

**Figure 5:**
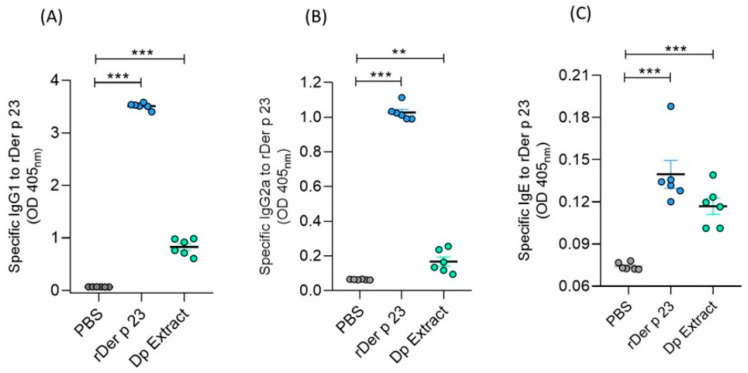
Serum levels of specific antibodies in mice. Microtiter wells coated with rDer p 23 (solid phase) were incubated with sera from PBS-, rDer p 23-, or *D. pteronyssinus* extract-treated mice (*X*-axis). Der p 23-specific IgG1 (**A**), IgG2a (**B**), and IgE (**C**) antibodies were measured by ELISA. Data are reported as individual ODs (405_nm_). The mean value of each group (*n* = 6 mice) and the bars representing the SEM are shown. These results were from two independently replicated experiments. The comparison was performed using the one-way ANOVA test and Dunnett’s post hoc analysis. ** *p* < 0.01, *** *p* < 0.001.

The administration of rDer p 23 induced significantly higher serum levels of specific IgG1, IgG2a, and IgE antibodies compared to mice treated with PBS (Figure 5A–C), with a ratio of IgG1/IgG2a of 3.42. Mice immunized with *D. pteronyssinus* extract showed significantly higher serum levels of IgG1, IgG2a, and IgE to rDer p 23, compared to those obtained with mice treated with PBS (Figure 5A–C).

### 2.7. rDer p 23 Induced a Type-2 Cytokine Profile

**Figure 6:**
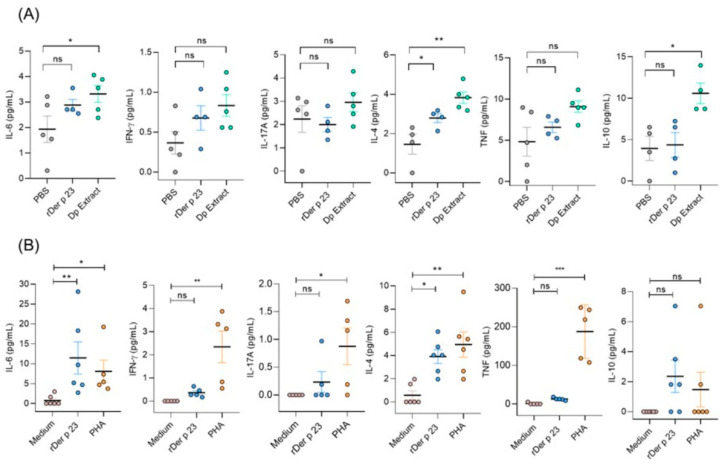
Cytokine profile in mice. (**A**) Cytokine levels in BALF of PBS-, Der p 23-, and D. pteronyssinus extract-treated mice. (**B**) Cytokine levels in splenocyte culture of rDer p 23-immunized mice stimulated in vitro with medium, rDer p 23, or PHA. Cytokine levels were determined by Cytometric Bead Array (CBA) and data are reported as individual concentrations (pg/mL). The mean value of each group (*n* = −4–6 mice) and the bars representing the SEM are shown. The comparison was performed using the one-way ANOVA test and Dunnett’s post hoc analysis. * *p* < 0.05, ** *p* < 0.01, *** *p* < 0.001, ns: not significant.

To evaluate the local and systemic immune responses induced by rDer p 23, cytokine levels were measured in BALF and splenocyte culture supernatants after in vitro stimulation with medium, rDer p 23, or phytohemagglutinin (PHA). Mice treated with rDer p 23 showed significantly higher levels of IL-4 in the BALF than PBS-treated mice and non-significant differences were found with the other cytokines tested. In mice immunized with *D. pteronyssinus*, extract levels of IL-4, IL-6, and IL-10 were significantly higher than those in mice treated with PBS (Figure 6A). These results agree with those found in splenocyte culture supernatants, where mice immunized with rDer p 23 and restimulated in vitro with rDer p 23 had a higher production of IL-4 and IL-6 than those stimulated with medium (Figure 6B).

### 2.8. rDer p 23 Did Not Induce Changes in Regulatory T Cell Frequency

**Figure 7:**
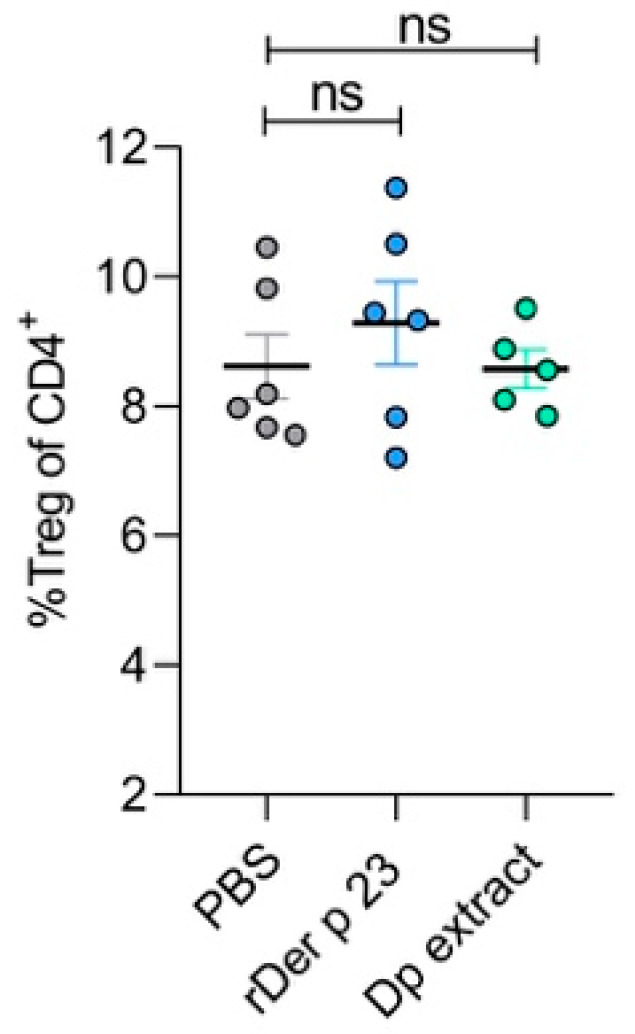
Analysis of spleen regulatory T cells. Statistical analysis of percentage of Tregs in the spleen of PBS-, rDer p 23-, and *D. pteronyssinus* extract-treated mice. The mean value of each group (*n* = 5–6 mice) and the bars representing the SEM are shown. ns: not significant.

This analysis showed that rDer p 23 administration did not induce changes in the frequencies of Tregs in in the spleen (Figure 7). The absence of significant production of Treg suggests that rDer p 23 and *D. pteronyssinus* extract did not affect Treg populations under our experimental conditions, and suggests a proinflammatory mechanism supporting the allergenic potential of rDer p 23.

### 2.9. rDer p 23 Exhibits High IgE Reactivity in Asthmatic Patients

**Figure 8:**
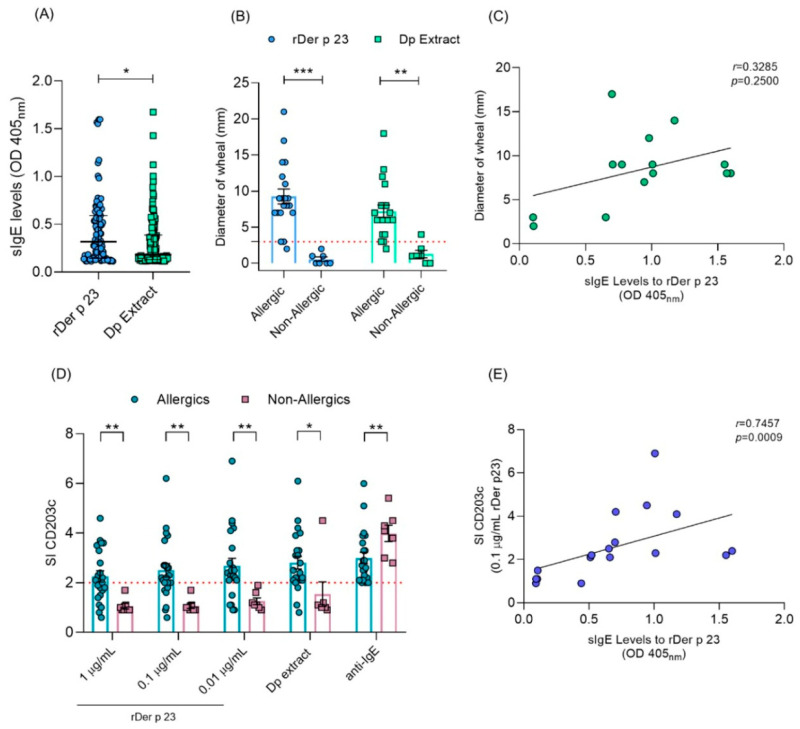
IgE reactivity to rDer p 23 and *D. pteronyssinus* extract in humans. (**A**) sIgE levels (OD 405 nm) to rDer p 23 (*n* = 81) and *D. pteronyssinus* extract (*n* = 111) in asthmatic patients with positive IgE values. (**B)** Diameter of wheal (mm) in allergic (*n* = 22) and non-allergic (*n* = 7) subjects by SPT. The red dashed line indicates cut-off point (**C**). Correlation analysis between sIgE levels to rDer p 23 and diameter of wheal by SPT. (**D**) Stimulation index (SI) (*y*-axis) of CD203c marker in allergic (*n* = 22) and non-allergic (*n* = 7) subjects by BAT. rDer p 23 (0.01, 0.1, and 1 μg/mL), *D. pteronyssinus* extract (1 μg/mL) (*x*-axis). The red dashed line indicates an SI ≥ 2.0 cut-off point for the test to be considered positive. (**E**) Correlation analysis between sIgE levels to rDer p 23 and the CD203c SI marker in BAT. The Mann–Whitney U test was used to assess differences in median sIgE levels, diameter of wheal, and CD203c SI between groups. Spearman’s test was used for correlation analysis between sIgE levels vs. CD203c SI and diameter of wheal. * *p* < 0.05, ** *p* < 0.01, *** *p* < 0.001.

The characteristics of the participants in this study are shown in Table 1. A total of 50.3% of patients with asthma were sensitized to rDer p 23 and 68.9% to *D. pteronyssinus* extract. In addition, among patients sensitized to *D. pteronyssinus* extract, 67.5% were sensitized to rDer p 23. The median IgE level to rDer p 23 (0.32 OD) was significantly higher than *D. pteronyssinus* extract (0.18, OD) (Figure 8A). Asthma-related biomarkers showed median fractional exhaled Nitric Oxide (FeNO) levels of 43 ppb and a blood eosinophil count of 240 cells/mm^3^. Lung function tests revealed median values for Forced Expiratory Volume in 1 Second (FEV_1_) and Forced Vital Capacity (FVC) of 79% and 91% of the predicted values, respectively, with an FEV_1_/FVC ratio of 0.75.

A total of 22 patients with allergic symptoms and 7 non-allergic subjects were evaluated by SPTs and BATs with rDer p 23 and *D. pteronyssinus* extract. It was found that 95.2% of SPTs in allergic patients were positive to rDer p 23 and *D. pteronyssinus* extract and showed an increased wheal diameter, whereas all non-allergic subjects obtained negative results to SPTs with rDer p 23, and only one subject obtained a positive result to *D. pteronyssinus* extract. These subjects showed a significantly smaller wheal diameter than allergic patients (*p* ≤ 0.0001) (Figure 8B). Correlation analysis between specific IgE (sIgE) levels and wheal diameter induced by rDer p 23 in allergic patients did not show a positive association (*r* = 0.3285; *p* = 0.2500) (Figure 8C). Also, it was observed that more than 80% of patients showed positive results in BATs in at least one of the evaluated concentrations of rDer p 23 and *D. pteronyssinus* extract, while in non-allergic subjects, rDer p 23 did not induce basophil activation under any of the conditions evaluated. The SI of the CD203c marker was significantly higher at all evaluated concentrations of rDer p 23 and *D. pteronyssinus* extract in allergic patients, compared to non-allergic subjects (*p* ≤ 0.0001) (Figure 8D). A positive association was found between rDer p 23 sIgE levels and SI CD203c in allergic patients (*r* = 0.7457; *p* = 0.009) (Figure 8E).

## 3. Discussion

HDM allergy is a leading cause of asthma and rhinitis worldwide [31,32]. Colombia is a tropical country with favorable environmental conditions that allow the perennial growth of mites throughout the year. Among them, *B. tropicalis* and *D. pteronyssinus* are the most commonly represented species in household dust samples from Cartagena [6,7]. *D. pteronyssinus* has 34 allergenic components officially recognized by the nomenclature subcommittee of the International Union of Immunological Societies (IUIS) (www.allergen.org/, accessed on 22 August 2025). Among these, Der p 23 is a clinically relevant allergen with an IgE reactivity frequency between 40% and 83% in HDM-sensitized patients around the world [16,27,28,33,34].

Previous studies identified rDer p 23 as a small (~8 kDa) protein by MALDI mass spectrometry, but the analysis by SDS-PAGE showed that the purified allergen migrated at ~14 kDa [28]. Our results show that rDer p 23 was successfully expressed in *E. coli* and purified to homogeneity, displaying an apparent molecular weight of ~17.5 kDa under reducing SDS-PAGE conditions. SEC-MALS revealed that rDer p 23 predominantly exists as a monomer (~12 kDa) in solution, with a minor proportion forming oligomers (~28 kDa) (Figure 1B). This difference may reflect a relevant characteristic: the Der p 23 sequence is rich in threonine and proline and thus closely resembles the PEST (proline, glutamate, serine, and threonine)-like regions typical of insect chitinases [28]. Proline-rich proteins typically exhibit reduced electrophoretic mobility, therefore migrating at a higher molecular mass on SDS-PAGE [35], as observed in previous studies [28,33].

The disappearance of the oligomeric state upon treatment with a reducing agent suggests the involvement of intermolecular disulfide bonds in oligomer formation (Figure 1B). This observation is consistent with previous structural studies of rDer p 23, which identified two disulfide bridges between Cys27 and Cys46, and between Cys56 and Cys69, as stabilizing elements of the molecule [27,33]. Furthermore, CD spectroscopy revealed that rDer p 23 adopts a predominantly random coil secondary structure (63.3%) and β-sheet (36,7%), with a strong negative ellipticity at 200 nm (Figure 1C). This structural profile aligns with earlier reports describing rDer p 23 as largely unstructured with a 61% random coil and 11% β-sheet [33]. Taken together, these findings corroborate and extend the previous characterization of rDer p 23, underscoring its importance as an allergen and validating the structural and biochemical reliability of the recombinant form produced at our institute for use in in vivo and in vitro experimental assays.

In the context of experimental models of allergic airway disease, both invasive LR measurements and WBP are essential and complementary tools for evaluating respiratory function and BHR [36]. In our model, both rDer p 23 and *D. pteronyssinus* extract significantly increased lung LR and Penh values at higher methacholine concentrations, indicating enhanced bronchoconstriction and airway sensitivity. These physiological changes are comparable to those observed in murine models exposed to HDM extract [37] and purified allergens such as Der p 1 and Der p 2 [9], as well as Blo t 12 and Blo t 2 [11,38], which promote airway hyperreactivity and inflammation. Penh value remains a useful surrogate marker of airway limitation when interpreted cautiously and in combination with invasive techniques [39]. Thus, the results obtained using both methods in this study strengthen the reliability of changes induced by rDer p 23 in lung function in this model.

Mice treated with rDer p 23 exhibited marked peribronchial and perivascular cellular infiltrates, albeit to a lesser degree than those exposed to the *D. pteronyssinus* extract. Furthermore, both rDer p 23 and the mite extract induced a comparable degree of goblet cell hyperplasia, a hallmark of allergy airway inflammation. This is the third *D. pteronyssinus* allergen in which an independent trigger of lung inflammation and structural airway changes have been demonstrated. The ability of this allergen to induce mucus secretion and epithelial changes suggest that specific HDM components play a direct role in asthma pathogenesis, as suggested from studies with Blo t 2 [11].

Exposure to rDer p 23 led to a pronounced eosinophilic infiltration in BALF, an important mechanism that contributes to Type-2 allergic airway inflammation. This finding aligns with previous reports demonstrating that other HDM allergens also induce eosinophil-dominant inflammation in murine asthma models [11,40,41]. The marked reduction in alveolar macrophages observed in *D. pteronyssinus* extract-treated mice reflects the loss of resident macrophages due to the intense inflammatory environment. This result was consistent with the recent findings of Feo-Lucas et al., who demonstrated that airway inflammation induced by HDM exposure triggered apoptosis and depletion of resident alveolar macrophages, followed by the recruitment of monocyte-derived macrophages and eosinophils [42]. The effect on these cell types induced by rDer p 23 was similar to but less pronounced than that observed with *D. pteronissynus* extract, suggesting that, although this purified allergen can elicit a relevant Type-2 inflammatory response, it lacks certain allergenic components present in the whole mite extract that potentiate inflammation. In addition, the increased frequency of T cells in mice exposed to rDer p 23 is in line with inducible BALF tissue by an IL-4- and IL-13-dependent mechanism in mice, as reported by Chua et al. [40], supporting the role of adaptive immunity in rDer p 23-mediated responses. These cellular dynamics collectively mirror those seen in mice treated with the complete *D. pteronyssinus* extract.

Aiming to better understand the early immunological events triggered by rDer p 23, we analyzed ILC-2 induced in the lungs of mice and found that rDer p 23 trends toward an increased proportion of a population of total ILCs and does not induce changes in the frequency of ILC-2. This could be a particular characteristic of Der p 23, but one of the limitations of this study is that we could not perform a functional experiment to confirm the profile of these cell populations. These findings suggest that Der p 23 induces a T2 allergic inflammation, whose pathophysiological mechanism may primarily involve the activation of signaling pathways associated with other immune cells, such as eosinophils.

Mice immunized with rDer p 23 exhibited significantly elevated serum levels of specific IgG1, IgG2a, and IgE antibodies compared to PBS-treated controls. The IgG1/IgG2a ratio of 3.42 suggests that rDer p 23 induces a polarized humoral response to the Type-2 phenotype that supports its allergenic activity. Specifically, mice exposed to rDer p 23 showed significantly higher levels of IL-4 in BALF, while other cytokines such as IL-6, TNF, IL-17A, and IFN-γ did not differ significantly. Moreover, splenocyte cultures from rDer p 23-immunized mice restimulated with rDer p 23 produced significantly higher IL-4 and IL-6 levels, supporting Th2 polarization. Altogether, our findings confirm that rDer p 23 is serologically reactive and suggest its capacity of orchestrating a cytokine microenvironment that underlies Type-2 inflammation in the lungs and spleen, where IL-4 is one of the main components. This activity could be confirmed by continuing other types of experiments, such as an IL-receptor blockade or knockout mouse model. As both pathways of activation on the target organ are shown, it could be speculated that, in asthmatic individuals responding primarily to Der p 23, an immunotherapy approach based on this allergen could be pertinent to evaluation. HDM allergy diagnosis based on Der p 1, Der p 2, and Der p 23 could increase the probability of the successful immunotherapy of mite-allergic individuals [43]. In this respect, exploring whether Der p 23 has the capacity to induce an IgG4 antibody in the model will be worth investigating.

Tregs play a key role in the maintenance of tolerance toward allergens in lung mucosa due to their ability to limit the development of airway inflammation through inhibiting the activity of effector inflammatory cells [44]. Our study found that rDer p 23 did not induce significant changes in the frequency of Tregs in the spleen. It has been demonstrated that the absence of Tregs increases the intensity and duration of inflammatory and allergy response, leading to an imbalance between the effector and regulatory mechanisms of immunity [45,46]. In this sense, there could be an additional mechanism driven by Der p 23 to explain this allergenic activity.

In Cartagena, Colombia, it has been demonstrated that there is a frequency of IgE sensitization to *D. pteronyssinus* extract of 64.6% [47], and clinical association with asthma [48], but the analysis of individual allergens in tropical regions is scarce. Several studies have shown a frequency of IgE sensitization to rDer p 23 in allergic patients of 74% (Italy, France, and Austria) [28], 83.7% (Spain) [16], 42% (Germany) [34], 75% (North America) [27], 54% (Thailand) [33], and 43% (Korea) [49]. The frequency of IgE sensitization to rDer p 23 among asthmatic individuals sensitized to *D. pteronyssinus* (67.5%) found in this study is consistent with a recent study where IgE sensitization in allergic patients from Colombia, Guatemala, and Costa Rica was 67% [50]. Our findings provide an important contribution to the understanding of IgE sensitization to rDer p 23 in tropical environments. However, we do not know which of the potential isoforms of Der p 23 [51] is the main inducer of sensitization in our region, the reason is probably that the representation of these isoforms in the environment is different between Europe and Latin America.

From a functional standpoint, our results demonstrate that patients with asthma symptoms exhibit great immune reactivity to rDer p 23 and *D. pteronyssinus* extract. Specifically, allergic individuals developed significantly larger wheal diameters in response to rDer p 23 and *D. pteronyssinus* extract in SPTs compared to non-allergic subjects. The BAT results further support the role of rDer p 23 in eliciting effector cell activation in allergic individuals. CD203c, a well-established basophil activation marker [50], showed a significantly higher SI at all tested concentrations in allergic subjects than in non-allergic subjects. These findings are in line with earlier studies showing that rDer p 23 induces basophil activation in a dose-dependent manner in sensitized individuals [28]. Importantly, the absence of basophil activation in non-allergic controls across the different conditions confirms the allergen-specific nature of the response. These results suggest that, in allergic patients, recognition of rDer p 23 by IgE antibodies is associated with the pathophysiological mechanisms that induce allergic symptoms.

No significant correlation was observed between rDer p 23-specific IgE levels and wheal diameter in SPTs (*r* = 0.3285; *p* = 0.2500), suggesting that factors beyond IgE levels, such as mast cell and basophil densities, skin reactivity, or local cytokine milieu, may modulate in vivo responses. This is consistent with prior findings where IgE levels did not always predict the magnitude of the skin reaction in SPTs [52]. Conversely, a strong positive correlation was found between rDer p 23-specific IgE levels and CD203c expression in BATs (*r* = 0.7457; *p* = 0.009), supporting the idea that in vitro basophil activation may better reflect IgE-mediated effector cell reactivity [53]. These observations highlight the complex interplay between sensitization and clinical reactivity in allergic disease. Collectively, these results emphasize the utility of combining multiple diagnostic approaches, including IgE quantification, SPTs, and BATs, to assess the allergen activity of particular HDM components.

In conclusion, our findings support the pro-allergenic role of rDer p 23 in the development of the pathological features of asthma and its ability to perpetuate allergic inflammation in the airways and to reveal the significant role of sensitization by Der p 23 in a tropical population.

## 4. Materials and Methods

### 4.1. Expression and Purification of Recombinant Der p 23

A codon-optimized nucleotide sequence of Der p 23 retrieved from GenBank XP_027193776.1, National Center for Biotechnology Information, Bethesda, MD, USA) without the signal peptide, was synthesized (GenScript, Piscataway, NJ, USA) for insertion into the multiple cloning site of a pET45b+ vector, flanked by HindIII and BamHI restriction sites and containing N-terminal His-Tag sequences for affinity purification. The recombinant plasmid was then used to transform *E. coli* (DE3) BL21 by electroporation, following the manufacturer’s instructions (MicroPulser^TM^ Bio-Rad, Hercules, CA, USA). Transformant colonies were selected on Luria broth agar plates containing 100 µg/mL ampicillin. Protein expression was induced with 0.1 mM IPTG for 5 h at 37 °C. For lysate preparation, cells were resuspended first in 20 mM NaH_2_PO_4_ and 300 mM NaCl (Native Binding Buffer pH 8.0) and sonicated for 5 cycles of 20 s in a Sonic Dismembrator 705 (Fisher Scientific, Hampton, NH, USA). For purification, the bacterial lysate was passed through a Ni-NTA column (Qiagen, Hilden, Germany) and eluted with NEB 250 mM imidazole, 50 mM NaH2PO4, and 300 mM NaCl, pH 8.0. Purified rDer p 23 was dialyzed in PBS (pH 7.4), using membranes with a molecular weight cut-off of 6000–8000 Daltons (Spectra/Por™, Spectrum Lab, cat. 132660, Waltham, MA, USA). For endotoxin removal, rDer p 23 was passed through a polymyxin B column (ToxinEraser^TM^ Endotoxin Removal, Cat. L00338 Genscript, Piscataway, NJ, USA) five times. Endotoxin levels were determined using a quantitative colorimetric assay based on the Limulus Amebocyte Lysate (LAL) reaction (ToxinSensor^TM^ Endotoxin Detection System, Cat. L00350. GenScript, Piscataway, NJ, USA). Optical density was measured at 545 nm on the Multiskan™ spectrophotometer (Thermo Fisher Scientific, Waltham, MA, USA).

### 4.2. Preparation of D. Pteronyssinus Extract

A whole *D. pteronyssinus* extract was prepared at the Institute for Immunological Research using cultured mites kindly provided by E. Fernández Caldas. Raw material was defatted by Soxhlet extraction in ethyl ether, then extracted in PBS buffer for 24 h at 4 °C and centrifuged. The supernatant was dialyzed in purified water using membranes with a molecular weight cut-off of 3500 Daltons (cat. 132720 Spectra/Por™, Waltham, MA, USA). Endotoxin levels in the *D. pteronyssinus* extract were removed using 1% Triton X-114 following a protocol described elsewhere [54]. Endotoxin levels were determined as described above.

### 4.3. Structural Analysis

#### 4.3.1. Size Exclusion Chromatography Coupled to Multi-Angle Light Scattering

SEC-MALS was performed with the ÄKTA FPLC system (Cytiva, Marlborough, MA, USA) using a Superdex 200, 10/300 GL column (Cytiva, Marlborough, MA, USA) and a flow rate of 0.3 mL/min. The system was connected to a miniDAWN Treos II MALS detector (Santa Barbara, CA, USA). rDer p 23 was concentrated using a 3000 Daltons cut-off membrane (Amicon Ultra-15; Merck Millipore, Burlington, MA, USA) in a buffer containing 50 mM Na2HPO4, 300 mM NaCl, pH 8.0. The data were evaluated using the software ASTRA software version 8.0 (Wyatt Technology, Santa Barbara, CA, USA).

#### 4.3.2. Stability Assay

Twelve percent SDS-PAGE analysis under reducing conditions was used to assess protein purity and stability upon its storage at 4 °C, for four weeks. In addition, rDer p 23 was prepared in non-reducing and reducing conditions using [Tris (2-carboxyethyl) phosphine hydrochloride] for evaluating the SDS-PAGE migration pattern and oligomeric state.

#### 4.3.3. Circular Dichroism Spectroscopy

CD spectroscopy of rDer p 23 was performed on a J-1500 spectropolarimeter (JASCO Corporation, Tokyo, Japan). Purified rDer p 23 was prepared in a concentration of 0.1 mg/mL in a 20 mM phosphate buffer (pH 8.0) containing 150 mM sodium fluoride. Measurements were performed at 20 °C using a 1 mm path length quartz cuvette. CD spectra were recorded in a 260 to 185 nm range with a 0.2 nm data pitch, 2 nm band width, and a scanning speed of 100 nm/min. Each spectrum was an average of 10 scans and was baseline-corrected by subtracting the spectrum of the buffer recorded under identical conditions. Data smoothing was executed through the Spectra Manager software suite, version 2.8 (JASCO Corporation, Tokyo, Japan) using a weighted means smoothing algorithm. Results were expressed as a mean residue ellipticity [θ] MRW at the given wavelength and the secondary structure was estimated using the BeStSel server version 2.15, JASCO Corporation, Tokyo, Japan [55].

### 4.4. Studies in Blalb/c Mice

#### 4.4.1. Allergic Airway Inflammation Model

Four-week-old female Balb/c mice were obtained from the National Institute of Health (Bogotá, Colombia), and six mice per cage were assigned to each group of treatment by simple randomization, housed under standard conditions in the animal facilities at the University of Cartagena with a controlled climate (22 +/− 3 °C, 45–55% relative humidity) and exposed to a 12 h/12 h light/darkness cycle. Animals were allowed to acclimatize for two weeks before use, and were fed with standard rodent diet (Ref. 5010, Rodent LabDiet, St. Louis, MO, USA) and drinking water ad libitum. Cage locations and sanitary care were equal for the three groups of treatments. Experiments were in accordance with the recommendations of the European Union regarding animal experimentation (Directive of the European Council 2010/63/EU) [56]. The research was approved by the Ethics Committee of the University of Cartagena (Protocol code 128, 14 November 2019). The mouse model of allergic airway inflammation was performed using a similar sensitization protocol as in a previous study [11]. No sample size calculation was performed. A sample size of at least four samples per group were in each experiment. Briefly, six- to eight-week-old female Balb/c mice were sensitized by i.p. injections on days 0, 7, and 14 with 20 µg of rDer p 23 (0.1 UE/µg of protein), *D. pteronyssinus* extract (0.04 EU/μg of protein), or PBS as a control plus 2 mg of aluminum hydroxide as an adjuvant (Imject^®^ Alum, Cat. 77161, Thermo Fisher Scientific, Waltham, MA, USA), followed by daily i.n. challenges on days 21, 22, and 23 under anesthesia with 100% sevoflurane (Ref. ACRG2L9117, Baxter, Deerfield, IL, USA). On day 24 and day 25, reactivity to methacholine challenge and euthanasia were performed, respectively. Investigators were not blinded in group allocations, except the pathologist in charge of histological analysis.

#### 4.4.2. Bronchial Reactivity to Methacholine Challenge

The functional capacity of the mice airways was evaluated by two methods. One method was by WPB (Buxco^®^, Holliston, MA, USA). For this purpose, Penh values were registered after nebulization with 200 μL of methacholine (cat. A2251-25G, Sigma-Aldrich, St. Louis, MO, USA) at different concentrations: 3.12, 6.25, 12.5, and 25 mg/mL. The other method was by FinePoint^TM^ Mouse RC System^TM^ (Buxco/Data Science International, St. Paul, MN, USA), which was used to evaluate RL and Cdyn in tracheostomized mice under sedation by intraperitoneal administration of a mix of xylazine 15 mg/kg (Erma, Funza, Cundinamarca, Colombia) and ketamine 40 mg/kg (Over^®^, San Vicente, Santa Fe, Argentina). The mice were intubated with a 0.19-inch tube and connected to mechanical ventilation and sensors to measure heart rate, respiratory rate, and blood pressure. RL and Cdyn were measured directly after nebulizing 10 µL of methacholine at the doses indicated above. Four mice died during this procedure; therefore, their data are absent from statistical analyses (one from PBS, one from Der p 23, and two from *D. pteronyssinus* extract treatments).

#### 4.4.3. Euthanasia and Collection of Biological Samples

Mice were euthanized by using a lethal dose of Euthanex^®^ (1.17 mg sodium pentobarbital/sodium diphenylhydantoin 0.15 mg, INVET, Palmer, PA, USA) ensuring compliance with the standards related to the animal’s well-being. A blood sample was taken from the abdominal aorta and deposited in tubes with 0.5 M EDTA. The samples were centrifuged at 4500 rpm for 5 min, and plasma was separated and stored at −80 °C for antibody measurement. BALF was harvested by flushing the lung airways via the trachea (3×) with 1 mL of ice-cold PBS containing a 1% complete protease inhibitor cocktail (Ref. P2714, Sigma-Aldrich, St. Louis, MO, USA). The spleen was obtained and placed in 3 mL of RPMI-1640 medium (Ref. 10-040-CV, Sigma-Aldrich, St. Louis, MO, USA) supplemented with 5% inactivated fetal bovine serum (FBS) (Ref. 16000044, Gibco, Grand Island, NY, USA). The right lung was removed and placed in 10% neutral-buffered formaldehyde for histological analysis (*n* = 6 per treatment group). For ILC-2 identification, the entire lungs were obtained from different groups (*n* = 6 per treatment group) and deposited in DMEM (Ref. 11995065, Gibco, Grand Island, NY, USA) supplemented with 10% FBS.

#### 4.4.4. Bronchoalveolar Lavage Fluid Processing for Cytokine and Inflammatory Cells Analysis

BALF was centrifuged at 1500 rpm for 5 min at 4 °C. The supernatant was used for measurement of Th1/Th2/Th17 cytokine levels by CBA (ref. 560,485 and ref. 558267, Becton Dickinson, Franklin Lakes, NJ, USA) using FACS-ARIA III flow cytometer (Becton Dickinson, Franklin Lakes, NJ, USA) with approximately 7000 events. The pellet was resuspended in 1 mL of 1X lysis buffer (cat. 555899, BD Pharm Lyse™, Franklin Lakes, NJ, USA for 5 min at 4 °C, centrifuged, and then resuspended in 60 µL of Stain Buffer. The mixture was divided into two aliquots of 30 µL each. One aliquot was incubated for 30 min with a mix of fluorochrome-labeled monoclonal antibodies (Table S2) for the identification of CD45^+^ inflammatory cells, such as alveolar macrophages (CD11b^low^CD11c^+^SiglecF^+^), eosinophils (CD11b^high^SiglecF^+^Ly6G^-^) and neutrophils (CD11b^high^SiglecF^+^Ly6G^+^) (Figure S3). The other aliquot was incubated with a mix of antibodies used as an isotype control (Table S3). The reading was performed on the FACS-ARIA III flow cytometer (BD™, Franklin Lakes, NJ, USA) with approximately 1000 events analyzed using Kaluza Analysis version 2.1 (Beckman Coulter, Brea, CA, USA).

#### 4.4.5. Identification of ILC-2 in Lungs

The lung samples for the identification and counting of ILC-2 were processed following the protocol based on [57]. Lung samples were sliced and digested with Lung Digestion Medium: DMEM supplemented with 10% FBS, 142.5 U/mL collagenase (Ref. 17104019, Gibco, Grand Island, NY, USA), 100 U/mL DNase I (Ref. 07470, STEMCELL, Vancouver, BC, Canada), and 100 U/mL penicillin/streptomycin (Ref. 15140-122, Gibco, Grand Island, NY, USA) for 30 min at 37 °C in a shaker. Cell suspension of each lung was obtained after centrifuging the cells with 36% Percoll (Ref. P1644-500ML, Sigma-Aldrich, St. Louis, MO, USA) for 10 min at 700 g and then incubating the cell pellet with 1% lysis buffer for 5 min. Cell viability was measured by 0.4% Trypan Blue (Ref. 15250-061, Gibco, Grand Island, NY, USA) exclusion using an automated cell-counter TC20 (BioRad, Hercules, CA, USA); in all cases, the viability was >80%. Lung cells were incubated for 30 min with a mix of fluorochrome-labeled monoclonal antibodies for the identification of cell surface markers (Table S4) and a mix of antibodies used as an isotype control (Table S5). The reading was performed on the FACS-ARIA III flow cytometer (Becton Dickinson, Franklin Lakes, NJ, USA) with approximately 1,000,000 events analyzed using Kaluza Analysis version 2.1 (Beckman Coulter, Brea, CA, USA). ILC-2 was identified as Lineaje^-^CD90.2^+^CD127^+^ST2^+^ (Figure S2).

#### 4.4.6. Determination of Cytokines in Splenocyte Cell Culture

A single cell suspension of a spleen was obtained after tissue homogenization and cell viability was measured by 0.4% Trypan Blue exclusion using an automated cell-counter TC20 (BioRad, Hercules, CA, USA); in cases when the cell viability was less than 80%, the sample was excluded from the cell culture (in one sample for stimuli with PHA, Der p 23 and PBS were not available). Splenocytes were cultured in RPMI-1640 medium supplemented with 1 mM sodium pyruvate (Ref. S8636, Sigma-Aldrich, St. Louis, MO, USA), 2 mM L-glutamine (Ref. G7513, Sigma-Aldrich, St. Louis, MO, USA), 100 U/mM penicillin-streptomycin (Ref. 15140-122, Gibco, Grand Island, NY, USA), and 10% heat-inactivated FBS. Cells (1 × 10^6^) were stimulated with 35 μg/mL rDer p 23, 2% PHA (Ref. 10576-015, Gibco, Grand Island, NY, USA), or medium RPMI 1640 supplemented and incubated at 37 °C and 5% CO_2_ for six days, or 72 h for PHA-stimulated cell cultures. Cell culture supernatants were stored at −80 °C until use. Th1/Th2/Th17 cytokine levels were determined by CBA (Ref. 560485, BD, Franklin Lakes, NJ, USA). Data analysis was performed using Flow Cytometric Bead Array software, version 3.0 (Becton Dickinson, Franklin Lakes, NJ, USA).

#### 4.4.7. Identification of Regulatory T Cells

Tregs were identified in the spleen cell suspension as CD3^+^CD4^+^CD25^+^Foxp3^+^ using fluorochrome conjugated antibodies (Table S6) and the gating strategy shown in (Figure S4), following the instructions of the anti-mouse Foxp3 staining kit (Ref. 00-5523-00, eBioscience, San Diego, CA, USA). Rat IgG2a kappa/PE isotype control (Ref. 12-4321-80, eBioscience, San Diego, CA, USA) was used as a staining control for the nuclear transcription factor FoxP3. Cells were identified in FACS Aria III (Becton Dickinson, Franklin Lakes, NJ, USA). Kaluza Analysis version 2.1 (Beckman Coulter, Brea, CA, USA) was used to analyze the results.

#### 4.4.8. Lung Histological Analysis

Lung histology was evaluated by a pathologist blinded for group assignments. The lung samples were embedded in paraffin and then cut into 4 μm thick sections and stained H/E (Ref. 6765015, Thermo Shandon, Pittsburgh, PA, USA) and PAS (Ref. HX99153846, Merck, Darmstadt, Germany). The sections were visualized by light microscopy to evaluate lung inflammation and mucus production. Lung inflammation was defined as the sum of the peribronchial and perivascular inflammation scores based on a modification of the 5-point scoring system described by Myuo et al. [58]. The degree of mucus production was determined by counting PAS-positive goblet cells using the 5-point scoring system described by Tanaka et al. [59]. Images were captured at a magnification of 10× and 40× on a light microscope connected to an ICC50 HD camera DM500 (Leica Microsystems, Wetzlar, Germany). Images were processed with Leica Application Suite software version 3.0.

#### 4.4.9. Determination of Antibody Serum Levels

sIgE, sIgG1, and sIgG2a levels were determined by ELISA. Microtiter plates were coated with 0.25 µg of rDer p 23 or 2.5 µg of *D. pteronyssinus* extract in sodium carbonate/bicarbonate buffer (pH 9.2) and incubated ON. Wells were then blocked with PBS-BSA 1%. Then, plasma samples were added at a dilution of 1:500 (IgG1 ELISA), 1:250 (IgG2a ELISA), or 1:6 (IgE ELISA), and incubated for 2 h at 37 °C in a humid chamber. After incubation with biotin-labeled anti-mouse IgG1 (cat.553441, BD Pharmigen™, Franklin Lakes, NJ, USA), and IgG2a (cat. 550332 BD Pharmigen™, Franklin Lakes, NJ, USA) for 2 h at 37 °C, and biotin-labeled anti-mouse-IgE (Ref. 13-5992-82, Clon 23G3, eBioscience™, San Diego, CA, USA). ON at 4 °C., alkaline-phosphatase streptavidin (cat. E2636, Sigma-Aldrich, St. Louis, MO, USA) was added to the wells and incubated for 1 h. 4-nitrophenyl phosphate (Ref. N2640, Sigma-Aldrich St. Louis, MO, USA,) was used as a substrate. The reaction was stopped with 3N NaOH and OD obtained at 405 nm in a spectrophotometer (Multiskan™, Thermo Fisher Scientific, Waltham, MA, USA). To increase the sensitivity of IgE ELISA, IgG antibody was depleted in plasma samples by incubation with Protein G Sepharose^®^ 4 Fast Flow (Ref. GE17-0618-01, Cytiva, Marlborough, MA, USA) before measuring specific IgE antibodies.

### 4.5. Human IgE Response to rDer p 23

#### 4.5.1. Study Population

The IgE response to rDer p 23 was investigated in 161 asthmatic patients (18–65 years old) from the project “Studies on the pathogenesis of asthma in the tropics: opportunities for knowledge generation and innovation in biomedicine. BPIN2020000100405”. From a population of 308 asthmatic patients in our database, we randomly selected those (161) for further analyses. Asthma was diagnosed by a physician and objectively confirmed by the reversibility of pulmonary obstruction after bronchodilator administration and FeNO > 25 ppb. Regarding the asthmatic population, the inclusion criteria were having a confirmed diagnosis of asthma by a physician from the research staff (following the 2024 GINA guidelines) [60], being ≥18 years old and residing in the department of Bolívar (Colombia) during the last 2 years. In addition, we had the results of spirometry, FeNO, blood eosinophils, and specific IgE levels to rDer p 23 and *D. pteronyssinus* extract. The exclusion criteria were as follows: being under 18 years of age, and having an autoimmune disease, immunodeficiency, malignancy, or any other chronic condition. Twenty-two subjects that underwent SPTs and BATs were those that, after 50 random phone calls on the database, agreed to attend a new visit and to give a blood sample for the BAT studies. In addition, SPTs and BATs were performed in seven non-allergic adults who were selected randomly from the aforementioned project without a history of allergic diseases, such as asthma, rhinitis, or atopic dermatitis, nor history of autoimmunity, immunosuppression, or active neoplasia. The project was approved by the University of Cartagena Ethics Committee (N°128 14 November 2019) and all participants gave written informed consent for serological testing.

#### 4.5.2. Evaluation of the IgE Reactivity to rDer p 23 by ELISA

In a 96-well microplate (Immunolon^®^ 4HBX, cat. 6404 Thermo Fisher Scientific, Waltham, MA, USA), 0.5 μg of recombinant allergen and 5 μg of mite extract were incubated in a carbonate/bicarbonate buffer pH 9.2 overnight (ON) at 4 °C. After washing the plate with PBS-Tween 20 at 0.1%, the wells were blocked with PBS- 1% BSA plus 0.02% sodium azide (blocking buffer) for 3 h at room temperature (RT) in a humid chamber. Then, sera diluted 1:5 in blocking buffer were added and incubated overnight at room temperature. Subsequently, anti-human IgE-alkaline phosphatase (cat. A3525, Sigma-Aldrich, St. Louis, MO, USA) diluted 1:2000 in conjugated buffer was added and incubated for 2 h at RT. The reaction was developed with 1 mg/mL of p-nitrophenyl phosphate (pNPP) (cat. N2640 Sigma-Aldrich, St. Louis, MO, USA) diluted in 0.5 M diethanolamine/MgCl2 pH 9.8 at a concentration of 1 mg/mL for 30 min. The optical density (OD) was read at 405 nm in a Multiskan™ spectrophotometer (Thermo Fisher Scientific, Waltham, MA, USA). For each ELISA experiment, a negative control serum (negative for all allergens and mite extracts) was used, along with a positive control (a serum positive for *B. tropicalis* extract in two-fold serial dilutions, from 1:5 to 1:320). Blocking buffer was used as the blank. Positive results were determined based on a cut-off calculated as the mean OD of six negative controls plus three standard deviations. Positive IgE sensitization to rDer p 23 and *D. pteronyssinus* extract was considered when an OD (405_nm_) ≥ 0.111 and ≥0.113, respectively, was obtained.

#### 4.5.3. Skin Prick Test

A SPT was performed by trained medical personnel. rDer p 23 (25 μg/mL) and *D. pteronyssinus* extract (25 ng/mL) diluted in glycerol were administered by the epicutaneous via the forearm of subjects. The appropriate concentration of rDer p 23 was obtained by titration at four different concentrations (12.5, 25, 35, and 50 μg/mL). In addition, histamine phosphate (10 mg/mL) was used as a positive control and glycerinated solution as a negative control. An SPT was considered positive for rDer p 23 or *D. pteronyssinus* extract when the wheal diameter was greater than 3 mm after subtracting the value obtained with the negative control. All subjects provided written informed consent for skin tests.

#### 4.5.4. Basophil Activation Test

A BAT was performed following the staining protocol from the Allergenicity kit (Beckman Coulter, cat. A17116) which consists of an optimized combination of three monoclonal antibodies (Table S7) for the identification of activated basophils using the CRTH2^+^CD203c^+^CD3^-^ strategy (Figure S5). For this, 50 µL of whole blood were mixed with 10 µL of the antibody cocktail and 10 µL of the stimuli at different concentrations: rDer p 23 (1.0, 0.1, and 0.01 µg/mL), *D. pteronyssinus* extract (1 µg/mL), anti-IgE (positive control) and PBS (negative control). The mix was incubated for 20 min at 37 °C protected from light and then 50 µL of the stopping solution (PBS/20 mM EDTA, 1860851 Thermo Scientific, Waltham, MA, USA) was added. Subsequently, the cells were lysed with 1 mL of 1% lysis buffer and centrifuged at 200× *g* for 5 min. The supernatant was discarded by aspiration and then the cellular pellet washed with PBS and centrifuged for 5 min at 200× *g*. Finally, the pellet was resuspended in 200 µL of PBS for reading by flow cytometry using the FACS-ARIA III equipment (Becton Dickinson, Franklin Lakes, NJ, USA). For reading, 500 basophils were acquired for each condition. Data analysis was performed using Kaluza version 2.1 software (Beckman Coulter, Brea, CA, USA). The upregulation of CD203c in response to allergens or controls was quantified as the stimulation index (SI), calculated as the ratio between the median fluorescence intensity (MFI) of the stimulated sample and unstimulated PBS. An SI ≥ 2.0 was considered positive.

#### 4.5.5. Statistical Analysis

SPSS version 25.0 (SPSS Chicago, IL, USA) and GraphPad Prism version 8.0 for Windows (GraphPad Software, San Diego, CA, USA) were used for data analysis. Age, IgE levels, FeNO, and Blood eosinophils in asthmatic patients were not normally distributed, and data normalization transformations were unsuccessful; therefore, they were reported as median and IQR. FEV_1_ and FVC were reported as mean values and standard deviation (SD). Continuous variables such as SI and wheal diameter were compared between allergic patients and healthy subjects using the Mann–Whitney U test. Spearman’s test was used for correlation analysis between IgE levels and SI in BATs and IgE levels and wheal diameter in SPTs. Two-way ANOVA with Tukey’s post hoc analysis was applied to compare data between groups in a BHR test. One-way ANOVA with Dunnet’s post hoc analysis was used to assess differences in BALF cell content, ILC-2, Treg cells, specific serum antibody levels, lung inflammation score, goblet cell score, and cytokine levels. For BALF, histology analysis, serum antibody levels, and WBP, there were neither exclusions of experimental units nor of data points during analysis.

## Figures and Tables

**Table 1:** Demographic and clinical characteristics of the study population.

Demographic Features	Asthma (N = 161)
Age (years), Median (IQR)	31 (23–45)
Males, n (%)	55 (34.2%)
Females, n (%)	106 (65.8%)
BMI (kg/m^2^), Median (IQR)	26 (23–29)
Socio-economic conditions	
Completed technical/university education, n (%)	103 (64.0)
Urban area of residency, n (%)	152 (94.4)
Low socio-economic status, n (%)	130 (80.7)
Access to sewer service, n (%)	146 (90.7)
Access to clean water service, n (%)	157 (97.5)
Access to garbage collection service, n (%)	157 (97.5)
Access to natural gas service, n (%)	160 (99.4)
IgE Sensitization	
Sensitization to *rDer p 23*, n (%)	81 (50.3)
Sensitization to *D. pteronyssinus* extract, n (%)	111 (68.9)
Sensitization to *rDer p 23* in *D. pteronyssinus* extract positives, n/N (%)	75/111 (67.5)
Biomarkers and lung function tests	
FeNO, Median (IQR) n	43 (25–80) 149
Blood eosinophils, Median (IQR) n	240 (134–400) 149
FEV_1_), Mean (SD) n	80 (16) 152
FVC, Mean (SD) n	93 (15) 152
FEV_1_/FVC, Median (IQR) n	0.75 (0.68–0.82) 152

BMI: Body Mass Index; FeNO: fractional exhaled Nitric Oxide; FEV_1_: Forced Expiratory Volume in 1 Second; FVC: Forced Vital Capacity; ppb: parts per billion; SD: standard deviation; IQR: interquartile range; N: total population; n: number of subjects with demographic and clinical features or with lung function test indicated.

## Data Availability

The original contributions presented in this study are included in the article/Supplementary Materials. Further inquiries can be directed to the corresponding author.

## Supplementary Materials

The following supporting information can be downloaded at: https://www.mdpi.com/article/10.3390/ijms262110765/s1.
