# Supplementary material for: Der p 23, A Frequent IgE Sensitizer in Humans, Induces Airway Inflammation in Mice
Source: Int J Mol Sci. 2025 Nov 5;26(21):10765. doi: 10.3390/ijms262110765 (PMC12609036; doi:10.3390/ijms262110765)
Supplement: Supplementary file 1 [file ijms-26-10765-s001.zip › Supplementary tables.pdf]

Table S1. Secondary structure content.

	rDer p 23 (CD)	*Crystal structure*
**Secondary structure**	**%**	**%**
α-helix	0	0
β-sheet	36.7	45.8
Other	63.3	54.2

For rDer p 23 the % was calculated based on the obtained circular dichroism data and using BeStSel server. For the crystal structure of Der p 23 (PDB-Code: 4ZCE) secondary structure content was calculated with PDBsum.

Table S2. Fluorochrome-labeled monoclonal antibodies for the identification of immune cells in bronchoalveolar lavage (BAL).

Antibody	Fluorochrome	Reference	Company
CD45	Alexa Fluor 488	53-0451-82	eBioscience
CD3e	BV 421	404-0031-82	eBioscience
CD11b	PE	12-0112-82	eBioscience
CD11c	PE-Cy7	25-0114-82	eBioscience
CD170 (Siglec-F)	PerCP/e-Fluor 710	46-1702-82	eBioscience
Ly6G	APC	17-5931-81	eBioscience
Fixable Viability Dye	eFlour 780	65-0865-14	eBioscience

Table S3. Fluorochrome-labeled monoclonal antibodies used as isotype control for the identification of immune cells BAL.

Antibody	Fluorocrhome	Reference	Company
CD45	Alexa Fluor 488	53-0451-82	eBioscience
CD3e	BV 421	404-0031-82	eBioscience
Rat IgG2b K	PE	12-4031-82	eBioscience
Armenian Hamster IgG	PE-Cy7	25-4888-82	eBioscience
Rat IgG2a k (CD170)	PerCP/e-Fluor 710	46-4321-80	eBioscience
Rat IgG2b K	APC	17-4031-82	Invitrogen
Fixable Viability Dye	eFlour 780	65-0865-14	eBioscience

Table S4. Fluorochrome-labeled monoclonal antibodies used for the identification of ILC-2 in lungs.

Antibody	Fluorochrome	Reference	Company
CD45	Alexa Fluor 488	53-0451-82	eBioscience
Lineage	eFlour 450	88-7772-72	eBioscience
CD127 (IL-7R)	PE	12-1271-82	eBioscience
ST2 (IL-33R)	PE-Cy7	25-9335-82	eBioscience
CD90.2	APC	17-0902-82	eBioscience
Fixable Viability Dye	eFlour 780	65-0865-14	eBioscience

Table S5. Fluorochrome-labeled monoclonal antibodies used as isotype control for the identification of ILCs in lungs.

Anticuerpo	Fluorocromo	Referencia	Empresa
CD45	Alexa Fluor 488	53-0451-82	eBioscience
Linaje	eFlour 450	88-7772-72	eBioscience
Rat/IgG2a, kappa	PE	12-4321-82	eBioscience
Rat/IgG2a, kappa	PE-Cy7	25-4321-81	eBioscience
Rat/IgG2a, kappa	APC	17-4321-81	eBioscience
Fixable Viability Dye	eFlour 780	65-0865-14	eBioscience

Table S6. Fluorochrome-labeled monoclonal antibodies used for the identification of Tregs in spleen.

Antibody	Fluorocrhome	Reference	Company
CD3e	BV 421	404-0031-82	eBioscience
CD4	APC	17-0042-82	eBioscience
CD25	PE/Cy5	15-0251-82	eBioscience
FoxP3	PE	12-5773-82	eBioscience
Fixable Viability Dye	eFlour 780	65-0865-14	eBioscience

Table S7. Fluorochrome-labeled monoclonal antibodies for the identification of activated basophils in peripheral blood.

Antibody	Fluorochrome	Reference	Company
CD3e	PE	555340	BD Pharmingen
CRTH2	APC	17-2949-42	eBioscience
CD203c	FITC	324614	Biolegend
