# Supplementary material for: Der p 23, A Frequent IgE Sensitizer in Humans, Induces Airway Inflammation in Mice
Source: Int J Mol Sci. 2025 Nov 5;26(21):10765. doi: 10.3390/ijms262110765 (PMC12609036; doi:10.3390/ijms262110765)
Supplement: Supplementary file 1 [file ijms-26-10765-s001.zip › Supplementary Figures.pdf]

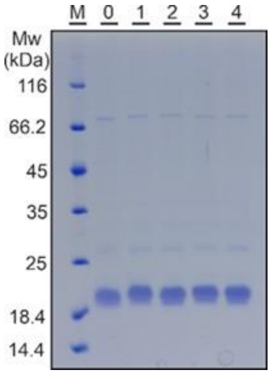

Figure S1. Stability assay. *Coomassie blue stained 12% (w/v) SDS polyacrylamide gel of rDer p 23 upon 4-week storage. Lane 0- 0 days, Lanes 1- 4 refer to 1-4 weeks of storage.*

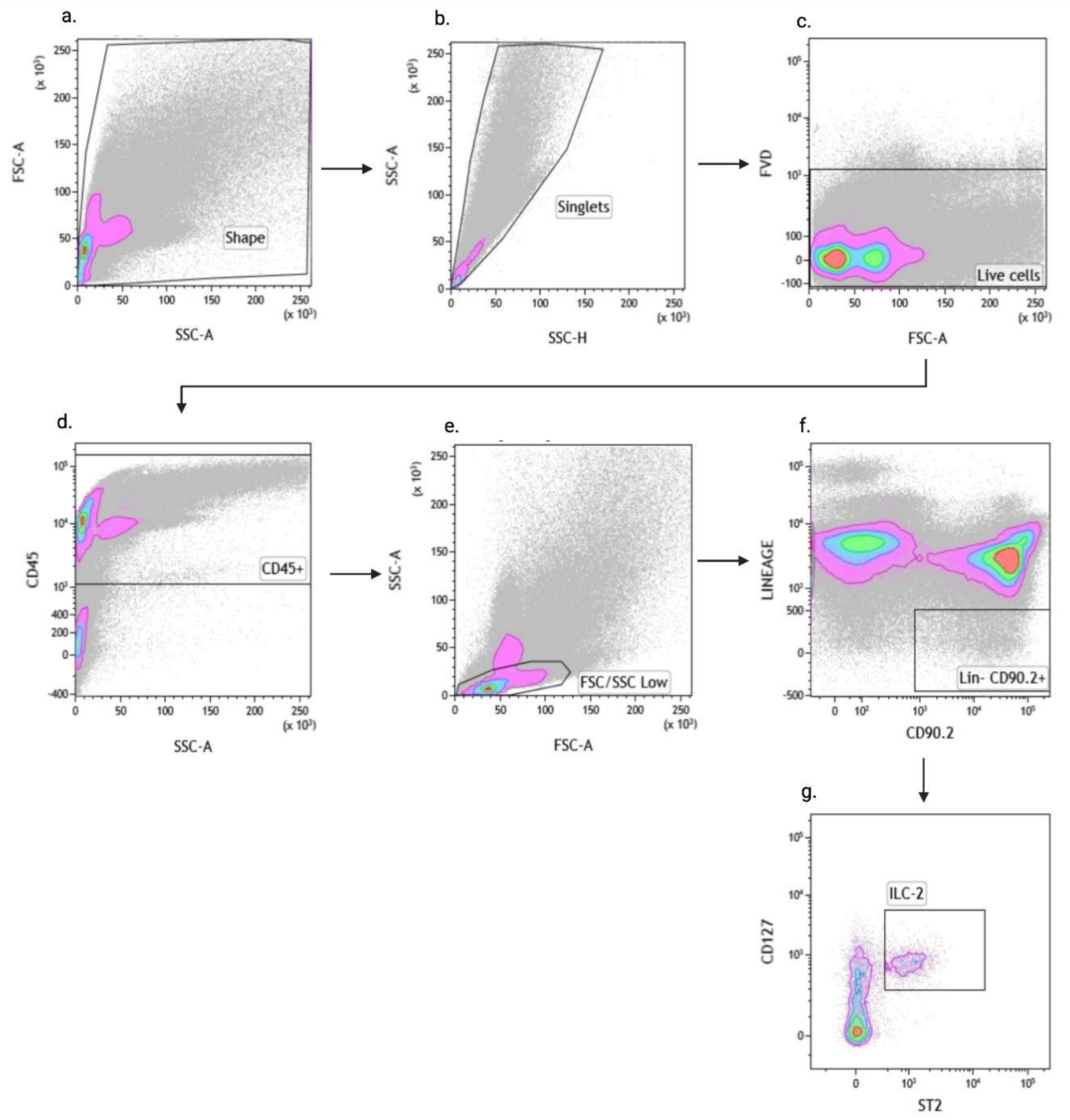

Figure S2. Gating strategy for identifying ILC-2 in lung. *Forward scatter (FSC) and side scatter (SCC) were used to include all cells (a) and remove cell debris and doublets (b). Within live cells that were selected using the viability marker Fixable Viability Dye (FVD) (c), the immune cells were identified as CD45^{+} (d). Lineage^{-} CD90.2^{+} cells were clustered as FSC/SSC^{low} (e, f). ILC-2 was identified as ST2^{+}CD127^{+} within the Lineage^{-}CD90.2^{+}(g).*

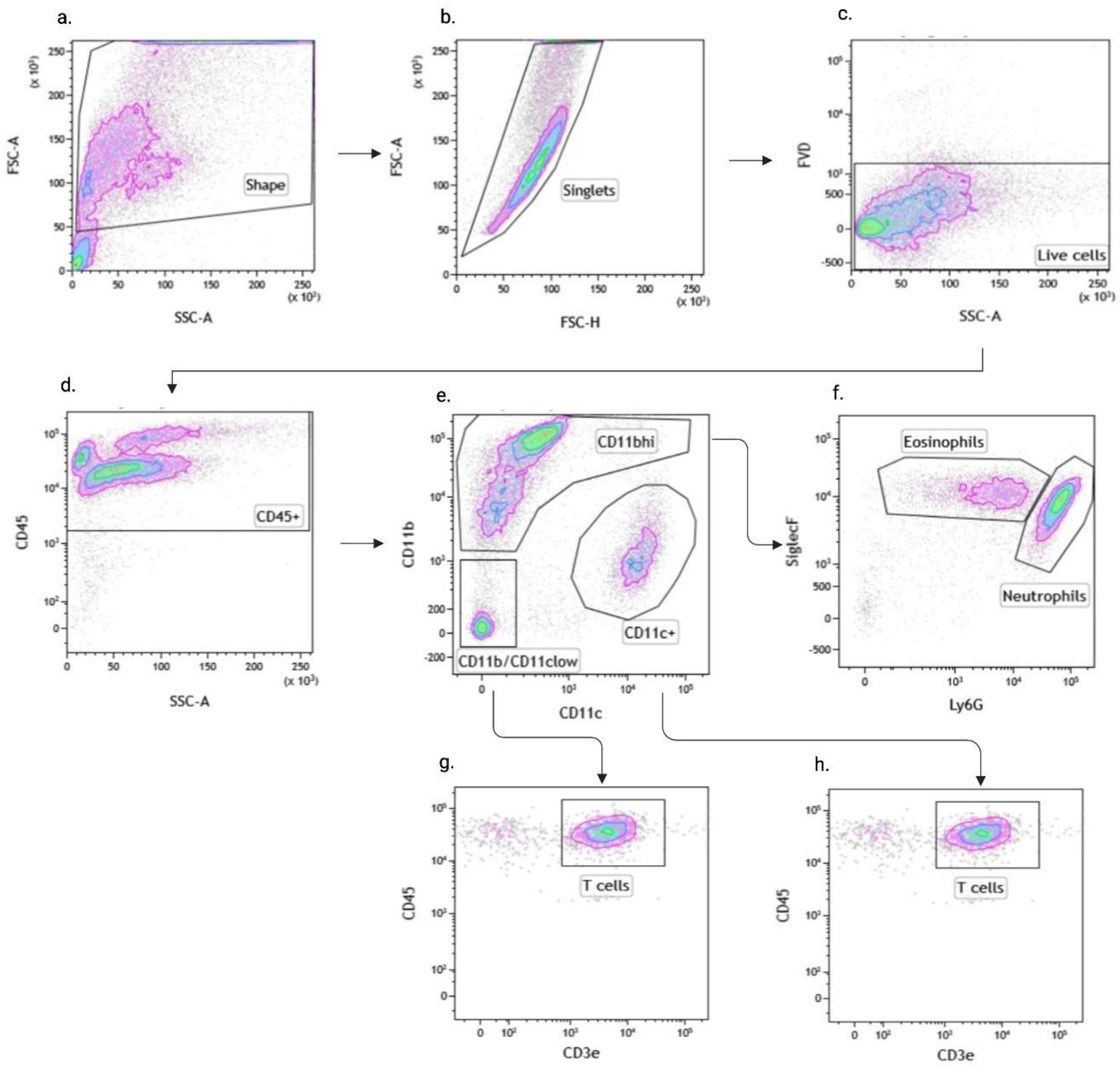

Figure S3. Gating strategy for identifying immune cells in BAL. *Forward scatter (FSC) and side scatter (SCC) were used to include all leukocytes (a) and to remove cell debris and doublets (b). Within live cells that were selected using the viability marker Fixable Viability Dye (FVD) (c), the immune cells were identified as CD45^{+} (d). Myeloid cells clustered as CD11b^{high} and CD11^{+} within CD45^{+} cells (e). Neutrophils were identified as Ly6G^{+} and eosinophils as SiglecF^{+} within the CD11b^{high} cell cluster (f). Alveolar macrophages were identified as CD11c^{+}SiglecF^{+} within the CD11c^{+} cell cluster (g). T cells were identified as CD45^{+}CD3^{+} cells within the CD11b/CD11c^{low} group (h).*

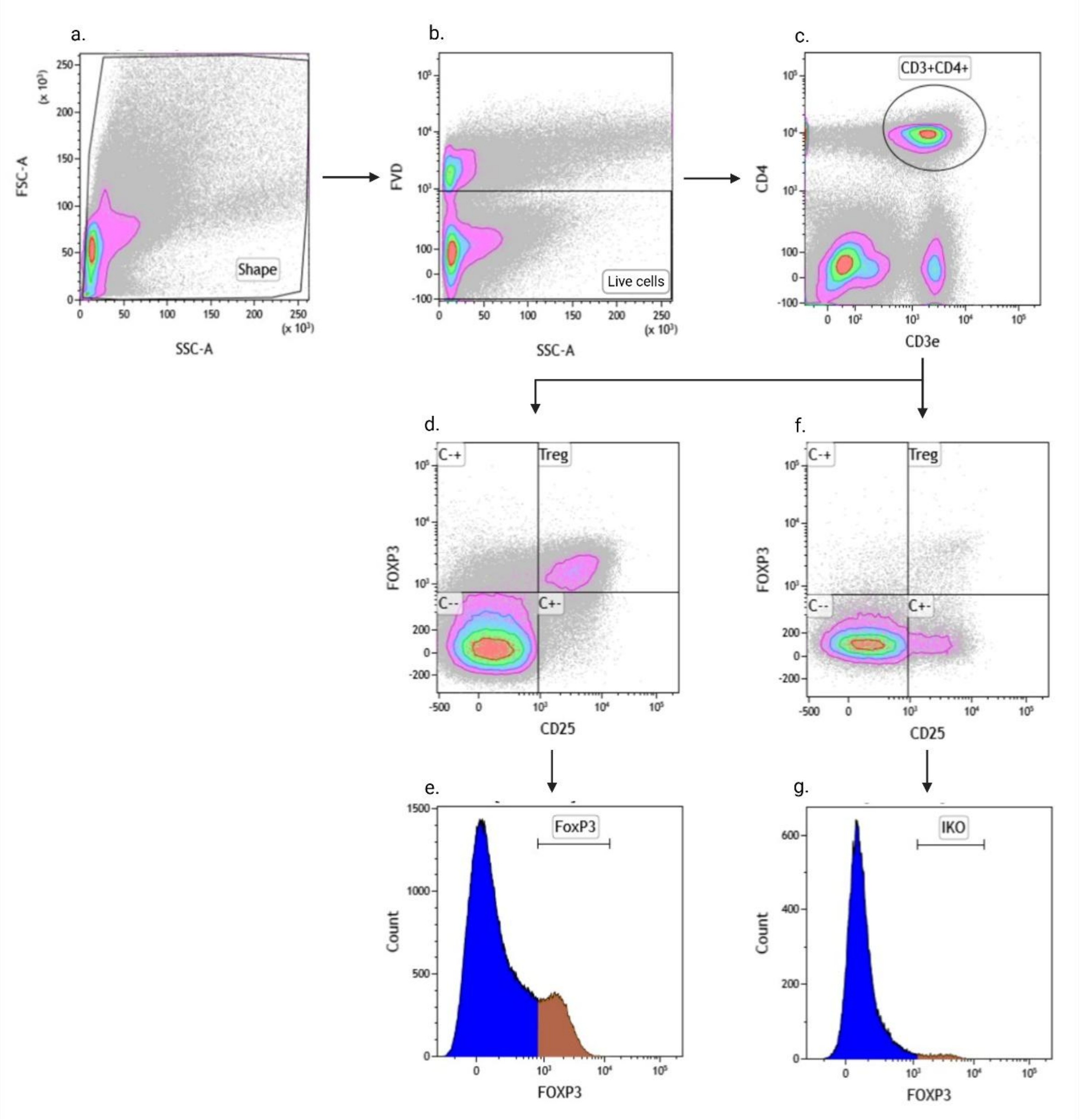

Figure S4. Gating strategy for identifying regulatory T cells in splenocyte suspensions. *Forward scatter (FSC) and side scatter (SCC) were used to include all cells (a). T cells were identified as $CD3^+CD4^+$ (b) within live cells that were selected using the viability marker Fixable Vibility Dye (FVD) (c). Regulatory T cells were identified by the expression of CD25 and the nuclear transcription factor FoxP3 (d, e,). Isotype control (IKO) was used as a staining control for the nuclear transcription factor FOxP3 (f, g).*

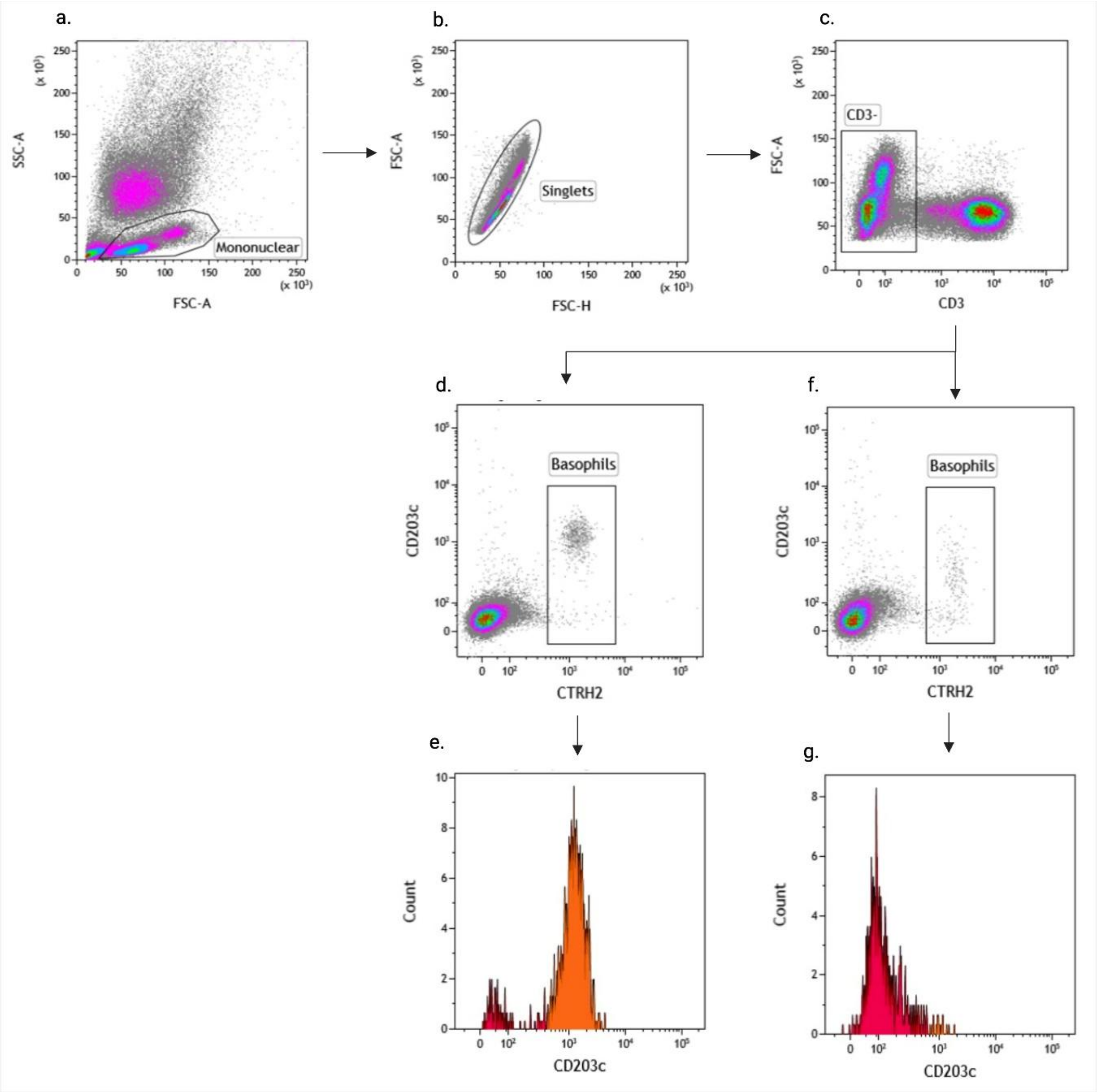

Figure S5. Gating strategy for identifying activated basophils in peripheral blood. *Forward scatter (FSC) and side scatter (SCC) were used to include all mononuclear leukocytes (a) and remove cell debris and doublets (b). CD3$^-$ cells were gated (c) to identify basophils with CRTH2$^+$ and CD203c$^+$ markers (d, f). Upregulation of the CD203c marker was used to assess basophil activation stimulated with allergens or anti-IgE (e) or not stimulated with PBS (g).*
